# Experience with isavuconazole in lung transplant recipients with *Aspergillus* isolation in respiratory tract specimens: A multicenter, observational study

**DOI:** 10.1016/j.jhlto.2025.100419

**Published:** 2025-10-29

**Authors:** Jose Tiago Silva, Francisco López-Medrano, Alicia de Pablo, Andrea Gutiérrez-Villanueva, Ana Fernández-Cruz, Ángela Cano, Elisa Ruiz-Arabi, Julián Torre-Cisneros, Arnau Monforte, Cristina Berastegui, Oscar Len, David Brandariz-Núñez, Dolores Sousa-Regueiro, Myrian Aguilar-Pérez, Mario Fernández-Ruiz, José Maria Aguado

**Affiliations:** a Unit of Infectious Diseases, University Hospital “12 de Octubre,” Instituto de Investigación Hospital “12 de Octubre” (imas12), School of Medicine, Universidad Complutense, Madrid, Spain; b Centro de Investigación Biomédica en Red de Enfermedades Infecciosas (CIBERINFEC), Instituto de Salud Carlos III (ISCIII), Madrid, Spain; c Unit of Lung Transplantation, Department of Pneumology, University Hospital “12 de Octubre,” Madrid, Spain; d Unit of Infectious Diseases, Department of Internal Medicine, Hospital Universitario Puerta de Hierro-Majadahonda, Instituto de Investigación Sanitaria Puerta de Hierro, Majadahonda, Spain; e Unit of Infectious Diseases, Hospital Universitario Reina Sofía, Instituto Maimónides de Investigación Biomédica de Córdoba (IMIBIC), School of Medicine, University of Córdoba, Córdoba, Spain; f Department of Infectious Diseases, Hospital Universitari Vall d′Hebron, Barcelona, Spain; g Unit of Lung Transplantation. Hospital Universitari Vall d′Hebron, Barcelona, Spain; h Centro de Investigación Biomédica en Red en Enfermedades Respiratorias (CIBERES), Instituto de Salud Carlos III (ISCIII), Madrid, Spain; i Department of Pharmacy, A Coruña University Hospital Complex (CHUAC), A Coruña, Spain; j Unit of Infectious Diseases, A Coruña University Hospital Complex (CHUAC), A Coruña, Spain; k Department of Pneumology, Hospital Universitario Puerta de Hierro-Majadahonda, Madrid, Spain

**Keywords:** isavuconazole, lung transplantation, solid organ transplantation, invasive pulmonary aspergillosis, tracheobronchial aspergillosis

## Abstract

**Background:**

*Aspergillus* is a life-threatening opportunistic pathogen in lung transplant recipients (LuTRs). Isavuconazole (ISA) is a broad-spectrum triazole with favorable pharmacokinetics and safety profile. The clinical experience with ISA in LuTRs, however, is still limited.

**Methods:**

We performed an ambispective observational study from June 2019 to June 2024 in 5 Spanish centers. Adult LuTRs with isolation of *Aspergillus* spp. in respiratory tract specimens and indication of ISA therapy (either for the treatment of invasive pulmonary aspergillosis [IPA] or tracheobronchial aspergillosis [TBA] or for preemptive therapy) were eligible. The safety outcome comprised the occurrence of treatment-emergent adverse events (trAEs) and the need for modification of tacrolimus dose. Effectiveness outcomes included clinical, mycological, and radiological response at the end of treatment (EoT).

**Results:**

Overall, 50 LuTRs were included. ISA was administered for a median duration of 101 days (interquartile range: 27-161) for the treatment of IPA (18 [36.0%]) or TBA (27 [54.0%]) and as preemptive therapy (5 [10.0%]). The incidence rates of any trAE and trAE requiring premature ISA discontinuation were 24.0% and 10.0%, respectively. Most patients (35 [70.0%]) completed the planned course of therapy. Tacrolimus daily dose was reduced by a median of one-third in half of the patients (26/48 [54.2%]). Clinical, mycological, and radiological responses at EoT among were achieved in 66.7%, 84.2%, and 64.3% of evaluable patients, respectively. Aspergillosis-attributable mortality at the end of follow-up was 12.0%.

**Conclusions:**

ISA was well tolerated in LuTRs and proved to be effective for the treatment of invasive *Aspergillus* syndromes.

## Background

Every year, up to 4,600 lung transplant (LuT) procedures are performed worldwide.^1^ LuT is the optimal lifesaving treatment for end-stage lung diseases, such as chronic obstructive pulmonary disease, interstitial lung disease, or cystic fibrosis.^1^, ^2^ Advances in donor selection and allocation, surgical techniques, perioperative care, and long-term patient management have contributed to improve the survival and quality of life of LuT recipients.^1^, ^3^ Nonetheless, complications related to the lifelong use of immunosuppression, such as opportunistic infections, still take a heavy toll on this population in terms of morbidity and mortality.^4^, ^5^, ^6^ Invasive fungal infection (IFI) constitutes one of the most severe opportunistic infection after LuT, with *Aspergillus* spp. as the most common agent.^7^, ^8^ The clinical presentation of invasive *Aspergillus* syndromes comprises invasive pulmonary aspergillosis (IPA), with involvement of the lung parenchyma, and tracheobronchial aspergillosis (TBA), in which the infection is restricted to the trachea and bronchi.^9^, ^10^

Therapeutic options for IPA in LuT recipients are limited. Voriconazole (VORI), which is recommended as the first-line treatment,^11^, ^12^ has a significant potential for drug-drug interactions (DDIs) with calcineurin inhibitors and other immunosuppressive agents, requires therapeutic drug monitoring (TDM) due to interindividual variability secondary to polymorphisms in the *CYP2C19* gene and narrow therapeutic window, and is commonly associated with treatment-emergent adverse events (trAEs) such as liver toxicity, visual disturbances, or skin cancer.^13^ On the other hand, liposomal amphotericin B (L-AmB)—typically used as alternative or salvage therapy—may cause dose-limiting renal toxicity, hypokalemia, and hypomagnesemia.^14^, ^15^

Isavuconazole (ISA) is a second-generation broad-spectrum triazole with in vitro activity against yeasts, dimorphic fungi, and molds. ISA shows excellent oral bioavailability and predictable pharmacokinetics (PK), good safety profile, and lower incidence of trAEs as compared to VORI. Organ diffusion, including the central nervous system (CNS), is good. In addition, the potential for DDIs is not as meaningful as with other triazoles.^16^, ^17^, ^18^

Despite recent reports on the safety and effectiveness of ISA in solid organ transplantation (SOT),^19^, ^20^, ^21^ including PK studies,^22^, ^23^ the available experience in the LuT setting is still scarce. Therefore, we have performed a multicenter study to gain insight into the real-life use of ISA for the treatment of IPA and TBA in LuT recipients, including the preemptive therapy upon isolation of *Aspergillus* spp. from a respiratory tract specimen.

## Patients and methods

### Study design and setting

We performed an ambispective observational cohort study in 5 Spanish centers with extensive experience in LuT. The study was performed in agreement with the ethical standards laid down in the Declarations of Helsinki and Istanbul. The study protocol was approved by the ethics committee of the Hospital Universitario “12 de Octubre” (CEIm no. 18/414) and by each of the remaining centers, as required. The protocol was also evaluated by the Spanish Agency of Medicines and Medical Devices, which granted approval as a postauthorization safety study (JAG-ISA-2018-01).

Consecutive LuT recipients aged ≥18 years who received ISA for at least 24 hours following the diagnosis of IPA or TBA or as preemptive therapy due to the isolation of *Aspergillus* spp. in a respiratory tract specimen from June 2019 to June 2024 were considered eligible. Both intravenous and oral prescriptions were considered. The use of ISA as prophylaxis or empirical therapy (i.e., in the absence of a diagnosis of IPA or TBA or a positive respiratory tract culture for *Aspergillus* spp.) was excluded. Only proven or probable diagnosis of IPA and TBA according to the updated criteria proposed by the European Organization for Research and Treatment of Cancer and the Mycoses Study Group were considered.^24^ Investigators at each study site independently evaluated and confirmed patient eligibility and compliance with inclusion criteria.

The safety outcome comprised the incidence rates of any trAE, trAE requiring discontinuation of ISA therapy, and modifications of tacrolimus daily dose to maintain target trough level. Effectiveness outcomes included the clinical, mycological, and radiological response for cases of IPA at the end of treatment (EoT).

By means of a standardized case report form, the following variables were collected demographics; underlying end-stage lung disease; pre- and post-transplant chronic comorbidities; maintenance immunosuppression regimen; occurrence of acute graft rejection and chronic lung allograft dysfunction (CLAD); previous antifungal treatment; indication for the prescription of ISA (targeted treatment or preemptive therapy); type of invasive *Aspergillus* syndrome (site and diagnostic category); isolated *Aspergillus* species; length of therapy with ISA; use of monotherapy or combination therapy; TDM data for ISA if available; occurrence and grade of trAEs; changes in the corrected QT interval (cQT) during the first 2 weeks of ISA therapy; tacrolimus dose adjustments and TDM; clinical, microbiological, and radiological response at the EoT; and all-cause and aspergillosis-attributable mortality. All patients were followed up for at least 2 weeks from the discontinuation of ISA therapy or to the date of death if occurred earlier. All data were recorded and managed in a secure database.

### Study definitions

The diagnosis of IPA or TBA was established according to the European Organization for Research and Treatment of Cancer and the Mycoses Study Group criteria.^24^ Preemptive therapy was defined as the administration of ISA in LuT recipients with isolation of *Aspergillus* spp. in respiratory specimens with no clinical, radiological, or bronchoscopic evidence of invasive syndrome.^25^ The definitions of clinical, mycological, and radiological response were adapted according to the criteria applied in the phase 3 pivotal randomized clinical trial (SECURE), whose results led to the approval of ISA for the treatment of invasive aspergillosis.^26^ Clinical response was defined as the complete or partial resolution, at EoT, of all attributable signs and symptoms, including the resolution of bronchoscopic findings in cases of TBA. Mycological response was defined as the proven eradication of *Aspergillus* spp. in the respiratory tract samples obtained at EoT. Radiological response required the resolution or improvement of at least 50% of the radiological lesions attributable to IPA in the thoracic imaging performed closest to the EoT. Patients who received ISA as preemptive therapy were excluded from the estimation of clinical and radiological response rates, whereas those with diagnosis of TBA in the absence of findings suggestive of IPA in the thoracic imaging at diagnosis were excluded from the assessment of radiological response. Premature discontinuation was defined as the withdrawal of ISA before completing the planned length of therapy. The Common Terminology Criteria for Adverse Events version 5.0 criteria were applied for the grading of trAEs.^27^ Combination therapy was defined by the concomitant use of ISA and an additional antifungal agent active against *Aspergillus* for at least 72 hours. Estimated glomerular filtration rate was assessed using the abbreviated Modification of Diet in Renal Disease (MDRD-4) equation.

### Statistical analysis

Continuous variables were summarized by the mean ± standard deviation or the median with interquartile ranges (IQR). Categorical variables were summarized using absolute counts and percentages. Repeated measurements across time points were compared using paired parametric or nonparametric tests (Student’s *t*-test for paired samples or Wilcoxon signed-rank test), as appropriate. Statistical analysis was performed with SPSS version 23.0 (IBM Corp, Armonk, NY).

## Results

### Study population and baseline characteristics

We included 50 LuT recipients, whose clinical features are detailed in Table 1. Thirty patients (60.0%) were prospectively enrolled, whereas the remaining 20 (40.0%) were retrospectively included. Overall, there were no relevant differences according to the form of patient recruitment, with the exception of a lower prevalence of previous acute rejection and a higher proportion of cases of TBA as the indication for ISA therapy in the prospective subcohort (Table S1 in Supplementary Material). Mean age at the initiation of ISA therapy was 58.8 ± 10.1 years. The most frequent underlying condition was chronic obstructive pulmonary disease (25 patients [50.0%]) and usual interstitial pneumonia (11 [22.0%]). Most recipients had undergone double LuT (37 [74.0%]) and were receiving triple immunosuppression comprising prednisone (49 [98.0%]), tacrolimus (48 [96.0%]), and mycophenolate mofetil or mycophenolate sodium (30 [60.0%]). Well-established risk factors for IPA, such as previous acute rejection or CLAD, were present in 14 (28.0%) and 4 recipients (8.0%), respectively. The most common antirejection therapy consisted of corticosteroid boluses (13/14 [92.9%]).

**Table 1:** Demographics and Clinical Characteristics of the 50 LuT Recipients Included

Variable	
Age at transplantation, years [mean±SD]	56.6±12.3
Age at initiation of ISA therapy, years [mean±SD]	58.8±10.1
Male gender [*n* (%)]	39 (78.0)
Underlying end-stage lung disease [*n* (%)]	
COPD	25 (50.0)
Usual interstitial pneumonia	11 (22.0)
Primary pulmonary hypertension	6 (12.0)
Interstitial lung disease	5 (10.0)
Cystic fibrosis	2 (4.0)
Bilateral bronchiectasis	1 (2.0)
Pretransplant *Aspergillus* colonization [*n* (%)]	4 (8.0)
Type of LuT [*n* (%)]	
Single lung	13 (26.0)
Double lung	37 (74.0)
Previous LuT [*n* (%)]	1 (2.0)
Pretransplant comorbidities [*n* (%)]	
Diabetes mellitus	11 (22.0)
Chronic heart disease	7 (14.0)
Post-transplant complications [*n* (%)]	
Reintervention within the first month	9 (18.0)
Acute graft rejection^a^	14 (28.0)
Time interval to initiation of ISA therapy, days [median (IQR)]	343 (39-852.5)
CLAD^b^	4 (8.0)
Time interval to initiation of ISA therapy, days [median (IQR)]	1,060 (137-4,506.8)
Post-transplant antifungal prophylaxis [*n* (%)]^c^	47 (94.0)
Nebulized L-AmB	43 (86.0)
Nebulized ABLC	2 (4.0)
Caspofungin	2 (4.0)
Fluconazole	1 (2.0)
Induction therapy [*n* (%)]	
Basiliximab	35 (70.0)
Immunosuppressive regimen at initiation of ISA [*n* (%)]	
Corticosteroids	49 (98.0)
Daily prednisone dose or equivalent, mg [median (IQR)]	30.0 (12.5-40.0)
Tacrolimus	48 (96.0)
Trough level, mg/ml [median (IQR)]	10.7 (8.3-12.5)
Mycophenolate mofetil or mycophenolate sodium	30 (60.0)
Daily dose, g [median (IQR)]	2.0 (1.5-2.0)
Azathioprine	1 (2.0)

Abbreviations: ABLC, amphotericin B lipid complex; CLAD, chronic lung allograft dysfunction; COPD, chronic obstructive pulmonary disease; ISA, isavuconazole; IQR, interquartile range; L-AmB, liposomal amphotericin B; LuT, lung transplantation; SD, standard deviation.

a Thirteen patients received corticosteroid boluses for 3 days (total dose of 1,529 ± 540 mg), and 1 further patient was treated with extracorporeal photopheresis.

b One patient received antithymocyte globulin plus corticosteroid boluses, 1 further patient was treated with extracorporeal photopheresis, and the 2 remaining patients were maintained on the same immunosuppression.

c One patient received nebulized L-AmB plus fluconazole.

### Characteristics of IPA and previous antifungal treatment

Included patients received ISA for the treatment of IPA (18 [36.0%]) or TBA (27 [54.0%]), or as preemptive therapy (5 [10.0%]). In the IPA group, the infection was diagnosed in the native lung in only 1 patient (1/18 [5.6%]). Regarding microbiological features, *Aspergillus fumigatus* was the most common identified species (14 [28.0%]). Four (8.0%) patients had mixed infection by different species. The median interval since LuT to diagnosis was lower for TBA than for IPA (29 [IQR: 13-348] vs 353 [IQR: 196.8-662] days; *p*-value = 0.012). There were no cases of donor-derived infection, and the diagnosis of IPA or TBA was established de novo after transplantation in most patients (41/45 [91.1%]). The median time between LuT and the initiation of ISA therapy was 250.5 days (IQR: 25.8-568.8). Overall, 18 recipients (36.0%) were receiving a systemic agent with in vitro activity against *Aspergillus* when ISA was initiated, namely VORI (8 [16.0%]) or an echinocandin (8 [16.0%]). The most common reasons for prescribing ISA were the prevention of DDIs with immunosuppressive agents (15 [30.0%]), previous toxicity associated with VORI (11 [22.0%]), and oral step-down therapy from an echinocandin (6 [12.0%]) (Table 2).

**Table 2:** Indications and Characteristics of Courses of ISA Therapy (*n* = 50)

Variable	
Indication for antifungal therapy [*n* (%)]	
IPA	18 (36.0)
TBA	27 (54.0)
Preemptive therapy	5 (10.0)
Previous antifungal therapy [*n* (%)]	18 (36.0)
Voriconazole	7 (14.0)
Anidulafungin	5 (10.0)
Micafungin	2 (4.0)
L-AmB	2 (4.0)
Posaconazole	1 (2.0)
Voriconazole plus anidulafungin	1 (2.0)
Length of previous therapy, days [median (IQR)]	10 (6-17)
Isolated species [*n* (%)]	
*Aspergillus fumigatus*	14 (28.0)
*Aspergillus niger*	12 (24.0)
*Aspergillus terreus*	10 (20.0)
*Aspergillus flavus*	6 (12.0)
*Aspergillus lentulus*	1 (2.0)
*Aspergillus nidulans*	1 (2.0)
*A. fumigatus* and *A. lentulus*	1 (2.0)
*A. nidulans* and *A. terreus*	1 (2.0)
*A. flavus* and *A. terreus*	1 (2.0)
*A. fumigatus, A. terreus, A. flavus*, and *A. niger*	1 (2.0)
*Aspergillus* spp.	1 (2.0)
Not available	1 (2.0)
Reason for ISA therapy [*n* (%)]	
First-line therapy	15 (30.0)
Prevention of DDIs with immunosuppressive agents	15 (30.0)
Previous trAEs with voriconazole^a^	11 (22.0)
Pharmacokinetic optimization^b^	6 (12.0)
Salvage therapy due to refractory infection	1 (2.0)
Previous trAEs with fluconazole	1 (2.0)
Previous trAEs with anidulafungin	1 (2.0)
Time interval from LuT to initiation of ISA therapy, days [median (IQR)]	250.5 (25.8-568.8)
Initial mode of administration [*n* (%)]	
Oral	50 (100.0)
Type of antifungal regimen [*n* (%)]	
ISA monotherapy	37 (74.0)
Combination therapy	
Anidulafungin	7 (14.0)
Micafungin	6 (12.0)
Length of combination therapy, days [median (IQR)]	13 (9-20)
Length of ISA therapy, days [median (IQR)]	101 (27-161)

Abbreviations: DDI, drug-drug interaction; IPA, invasive pulmonary aspergillosis; ISA, isavuconazole; IQR, interquartile range; L-AmB, liposomal amphotericin B; LuT, lung transplantation; TBA, tracheobronchial aspergillosis; trAE, treatment-emergent adverse event.

a Liver toxicity (*n* = 5), acute kidney injury (*n* = 1), hallucinations (*n* = 1), severe dyspepsia, and intolerance not otherwise specified (*n* = 4).

b Oral stepdown from echinocandin therapy.

### Description of the treatment course with ISA

In all cases, ISA was administered orally, and mainly as monotherapy (37 [74.0%]). In the remaining 13 patients (26.0%), ISA was initially used in combination with an echinocandin. The median duration of antifungal combination therapy was 13 days (IQR: 9-20), whereas the median length of ISA therapy in the entire cohort was 101 days (IQR: 27-161). Seven patients (14.0%) were subjected to TDM for ISA (with 15 unique measurements) after a median interval since the initiation of therapy of 91 days (IQR: 41-117). The median ISA trough level was 5.00 mg/liter (IQR: 3.29-6.15) (Table 2).

### Safety outcomes

Twelve patients (24.0%) experienced at least 1 trAE during the course of ISA therapy. The most frequent events were acute kidney injury (4 [8.0%]) and mild hyperkalemia (4 [8.0%]), which did not require discontinuation of ISA and returned to normal levels once the daily dose of the calcineurin inhibitor was reduced. Five patients (10.0%) required premature discontinuation of ISA therapy due to trAEs: gastrointestinal symptoms (*n* = 3), liver toxicity (*n* = 1), and myopathy (*n* = 1). All trAEs requiring discontinuation resolved with no sequelae after a median of 6.5 days (IQR: 3.3-13.5) (Table 3). In 1 patient, ISA was resumed after 9 days, once the gastrointestinal symptoms had subsided. The patient subsequently completed a 3-month course without recurrence of toxicity. Interestingly, 1 patient who was initiated on ISA as salvage treatment due to severe VORI-induced liver toxicity also required discontinuation of ISA due to the progressive worsening of liver function tests.

**Table 3:** Safety Outcomes: Incidence of trAEs (*n* = 50)

Variable	
Occurrence of ≥1 trAE [*n* (%)]^a^	12 (24.0)
Acute kidney injury	4 (8.0)
Mild hyperkalemia (<6 mEq/liter)	4 (8.0)
Gastrointestinal symptoms (nausea and vomiting)	3 (6.0)
Elevation of liver enzymes	2 (4.0)
Myopathy	1 (2.0)
Skin cancer	1 (2.0)
trAE requiring premature discontinuation of therapy [*n* (%)]^b^	5 (10.0)
Time interval to remission from discontinuation, days [median (IQR)]	6.5 (3.3-13.5)
Reinitiation of therapy [*n* (%)]	1/5 (20.0)

Abbreviation: trAE, treatment-emergent adverse event.

a Three patients developed more than 1 trAE.

b Gastrointestinal symptoms (*n* = 3), liver toxicity (*n* = 1), and myopathy (*n* = 1).

Among the 7 evaluable patients (14.0%) with a 12-lead electrocardiogram performed twice in the first 2 weeks, no significant changes were observed in the cQT (444.3 ± 25.7 vs 445.3 ± 20.8 mseg in the first and second electrocardiogram, respectively).

**Figure 1:**
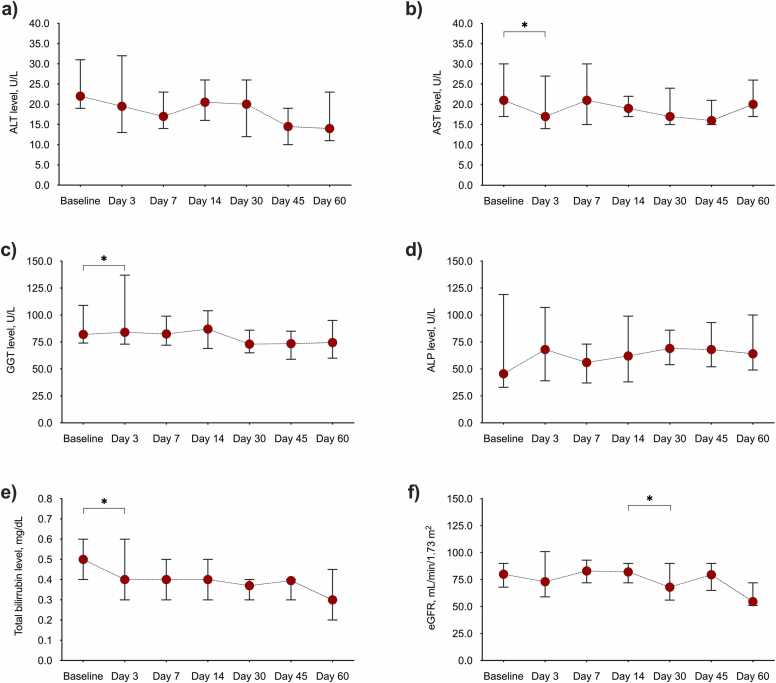
Laboratory parameters during the first 60 days of ISA therapy: (a) AST level, (b) ALT level, (c) GGT level, (d) ALP level, (e) total bilirubin, and (f) eGFR function. Circles and whiskers represent median values and 95% confidence intervals, respectively. *p-value < 0.05. ALP, alkaline phosphatase; ALT, alanine aminotransferase; AST, aspartate aminotransferase; eGFR, estimated glomerular filtration rate; GGT, gamma-glutamyl transferase; ISA, isavuconazole.

The evolution of liver function tests and eGFR is depicted in Figure 1. Aspartate aminotransferase and total bilirubin levels showed a significant decrease upon initiation of ISA therapy through day 3, whereas gamma-glutamyl transferase levels increased.

**Figure 2:**
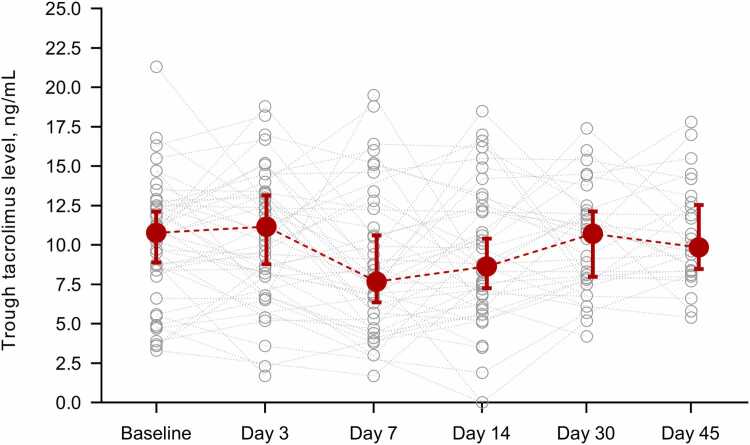
Results of therapeutic drug monitoring for tacrolimus during the first 6 weeks of ISA therapy. Gray lines represent individual values of trough tacrolimus levels, whereas red circles and whiskers represent median values and 95% confidence intervals, respectively. ISA, isavuconazole.

**Figure 3:**
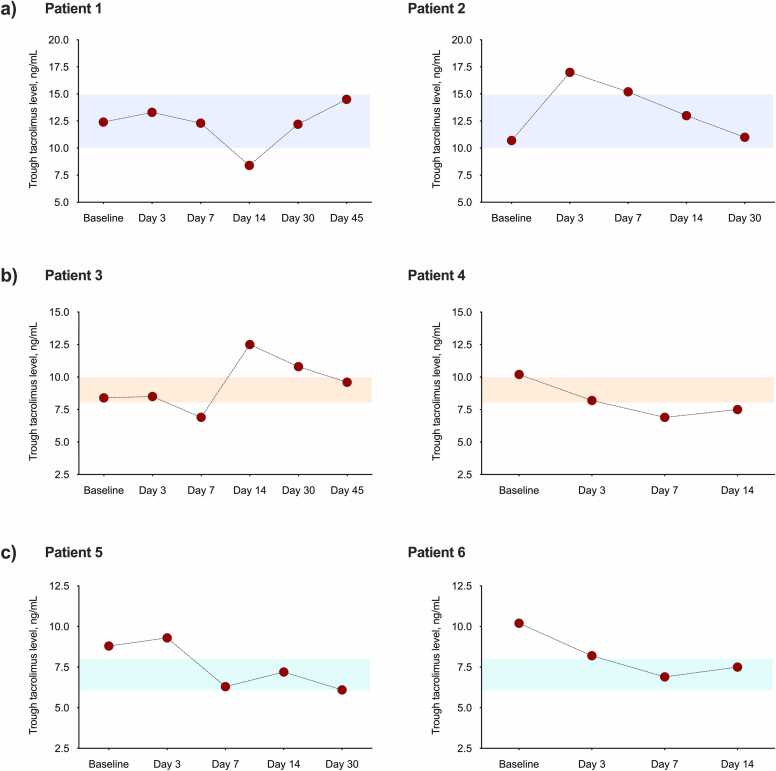
Longitudinal trajectories of TDM for tacrolimus in representative patients according to the post-transplant period and the commonly established target levels (colored areas): (a) first 90 days (tacrolimus target level: 10-15 ng/ml), (b) days 90 to 360 (tacrolimus target level: 8-10 ng/ml), and (c) beyond day 360 after transplantation (tacrolimus target level: 6-8 ng/ml). TBA, tracheobronchial aspergillosis.

Half of the patients (26/48 [54.2%]) required reduction of the daily dose of tacrolimus at the initiation of ISA therapy. The median dose reduction was 34.5% (IQR: 27.7-50.0). One-third (13/48 [27.1%]) subsequently required an increase in the dose by day 7. Overall, 223 unique TDM measurements for tacrolimus were performed during the first 6 weeks of ISA therapy. The median tacrolimus trough level during this period was 9.8 ng/ml (IQR: 6.8-12.5), with no significant differences in any of the repeated measures comparisons across monitoring points (Figure 2). Longitudinal TDM data in representative patients according to the post-transplant period and the target tacrolimus level are depicted in Figure 3.

### Effectiveness outcomes

The rates of clinical, mycological, and radiological response at EoT are detailed in Table 4. The planned course of ISA therapy was completed in 35 recipients (70.0%). The leading causes for stopping therapy were death not attributable to aspergillosis (4 [8.0%]), definitive discontinuation due to trAEs (4 [8.0%]), and aspergillosis-attributable death (3 [6.0%]). In 2 patients (4.0%), ISA was switched to caspofungin with nebulized VORI and posaconazole plus micafungin, respectively, due to IPA progression while on ISA therapy.

**Table 4:** Effectiveness Outcomes (*n* = 50)

Variable	
Completion rate of the planned course of ISA therapy [*n* (%)]	35 (70.0)
Reason for definitive discontinuation [*n* (%)]	
Death not attributable to aspergillosis	4 (8.0)
Premature discontinuation due to trAEs	4 (8.0)
Death attributable to aspergillosis	3 (6.0)
Therapeutic failure^a^	2 (4.0)
Progression of lung cancer	1 (2.0)
Transition to voriconazole for improving CNS diffusion	1 (2.0)
Clinical response at EoT [*n* (%)]^b^	30/45 (66.7)
Mycological response at EoT [*n* (%)]^c^	32/38 (84.2)
Radiological response at EoT [*n* (%)]^b^^,^^d^	9/14 (64.3)
All-cause mortality at the end of follow-up [*n* (%)]	13 (26.0)
Aspergillosis-attributable mortality^e^	6 (12.0)
Nonattributable mortality^f^	7 (14.0)

Abbreviations: CNS, central nervous system; EoT, end of treatment; IPA, invasive pulmonary aspergillosis; ISA, isavuconazole; trAE, treatment-emergent adverse event.

a Radiological progression of IPA.

b Patients who received ISA as preemptive therapy were excluded.

c The rate of mycological response was calculated on the number of evaluable patients with appropriate culture sampling at EoT.

d Patients with TBA in the absence of thoracic imaging findings at diagnosis were excluded. The rate of radiological response was calculated based on the number of evaluable patients with appropriate imaging follow-up at EoT.

e Three patients treated with ISA monotherapy eventually died of *Aspergillus*-related causes. One patient was switched to anidulafungin monotherapy due to ISA-induced hepatotoxicity and died 20 days later, 1 patient was switched to caspofungin and nebulized voriconazole due to IPA progression and died 119 days later, and 1 patient was switched to posaconazole and micafungin due to IPA progression and died 79 days later.

f COVID-19 (*n* = 2), liver and renal failure (*n* = 1), multifactorial respiratory failure (*n* = 1), hemorrhagic shock (*n* = 1), cardiac arrest (*n* = 1), and progression of lung cancer (*n* = 1).

Once excluded, those patients who received ISA as preemptive therapy (*n* = 5), clinical response rate at EoT was 66.7% (30/45) at EoT. Mycological response (i.e., culture conversion) was confirmed in 84.2% of evaluable patients (32/38), whereas radiological response was documented in 64.3% (9/14) of evaluable patients. In the subgroup with TBA, 21 (77.8%) and 20 (74.0%) out of 27 patients experienced clinical response and culture conversion at EoT, respectively. After a median follow-up of 100.5 days (IQR: 27.3-161), all-cause and aspergillosis-attributable mortality rates were 26.0% (13/50) and 12.0% (6/50), respectively. Three patients (6.0%) receiving ISA monotherapy eventually experienced IPA progression and died from attributable complications. Switching from ISA to alternative triazole-based regimens (VORI or posaconazole associated with an echinocandin) in 2 of them did not halt the progression of the infection.

## Discussion

We have performed the present multicenter study to describe the real-life experience with the use of ISA in LuT recipients for the treatment of invasive *Aspergillus* syndromes or for preemptive therapy after the isolation of *Aspergillus* spp. in the respiratory tract. The literature concerning the safety and effectiveness of ISA in the SOT populations remains limited, with only 2 previous studies that have specifically addressed this issue. The multicenter SOTIS (Solid Organ Transplantation and ISavuconazole) study included adult SOT recipients treated with ISA for mucormycosis or proven or probable invasive aspergillosis.^19^ Out of the 81 patients included, 71 had been diagnosed with an invasive *Aspergillus* syndrome and less than a third were LuT recipients, although the study did not provide subgroup analysis according to the SOT type. The authors reported 6- and 12-week clinical response rates for patients with invasive aspergillosis of 54.9% and 56.3%, respectively, whereas attributable mortality in the entire cohort by 12 weeks was 22.2%. About one-fifth of patients experienced at least 1 trAE, and 6.2% required permanent discontinuation of therapy.^19^ The ISASOT study was a Spanish non–comparative single-center prospective study that retrospectively recruited 53 SOT recipients (83.0% were LuT recipients) treated with ISA. The most frequent IFI was TBA, and *Aspergillus* spp. accounted for the majority of cases (81.1%). In the overall cohort, 27 patients (50.9%) achieved clinical cure at EoT, with a rate of culture conversion of 38.6%. All-cause mortality was 45.3%. The incidence of trAE was higher than in other series (49.1%), and 11.3% of patients required premature discontinuation.^21^ Table S2 details the results derived from our cohort compared to those reported from the SOTIS and ISASOT studies.

Our multicenter cohort lacks a control group treated with alternative triazoles, although previously published studies can serve for comparison. The Swiss Transplant Cohort Study included 70 SOT recipients with invasive aspergillosis mainly treated with VORI (84.6%) and reported a 12-week all-cause mortality (22.9%), similar to that observed in our study.^28^ The DiasperSOT study included 126 patients with a notable proportion of LuT recipients (42.8%). In accordance with the recruitment period (2010-2019), two-thirds of the patients received VORI. Noteworthy, a complete or partial clinical response by 12 weeks was observed in 54.6% of patients, close to the rate found in our study. In addition, 35.3% of the patients treated with VORI developed at least 1 trAE, which resulted in a discontinuation rate of 15.3%.^29^ Of note, these figures are higher than those derived from our experience (24.0% and 10.0%, respectively). The advantages of ISA over VORI in terms of safety and treatment completion rates have been confirmed in a recent post hoc comparative study of the SOTIS and DiasperSOT cohorts.^20^ Finally, a single-center study evaluating the effectiveness and tolerability of different regimens for antifungal prophylaxis after LuT reported a discontinuation rate significantly higher for VORI as compared to ISA (36% vs 11%, respectively), mainly due to the occurrence of hepatotoxicity and neurotoxicity.^30^ In this line, most trAEs in the present experience were mild in severity, with no cases of neurotoxicity or cQT-interval shortening, and all attributable symptoms resolved after a median of 6.5 days from withdrawal.

The management of DDIs between azoles and immunosuppressive agents is a crucial component of the treatment of IFI in the SOT population.^13^ ISA acts as a moderate inhibitor of the CYP3A4 isoenzyme,^31^, ^32^ and increases the area under the curve and the maximum serum concentration of tacrolimus by 2.3- and 1.4-fold, respectively.^33^ As expected, almost half of the patients required the daily dose of tacrolimus to be reduced by approximately one-third upon initiation of ISA therapy. Moreover, some patients required a further adjustment by increasing the tacrolimus dose at the end of the first week of therapy. Due to the significant interindividual variability in the impact of ISA on tacrolimus levels,^22^ TDM is mandatory to guide dose adjustments, as strongly recommended by clinical guidelines.^12^ The present experience suggests that, by this approach, the DDIs between ISA and tacrolimus are manageable.

Since ISA exhibits a linear PK and its bioavailability is less affected by food or genetic variability of drug metabolizing enzymes and drug transporters than other triazoles,^18^ routine TDM is not formally recommended.^15^ Nevertheless, the measurement of ISA levels may be advisable in selected cases of therapeutic failure, unexpected toxicity, isolates with elevated minimum inhibitory concentrations, or IFI affecting sanctuary sites such as the CNS.^15^ None of the LuT recipients who experienced trAEs in our study had ISA levels measured.

Three patients (6.0%) had IPA progression while on ISA monotherapy and eventually experienced attributable death. Although the underlying mechanisms are not entirely characterized, some studies have addressed the factors related to IPA presentation and host response that appear to negatively impact on patient’s outcome despite appropriate triazole-based therapy. A higher fungal burden at diagnosis, with pulmonary bilateral involvement or systemic dissemination (particularly to the CNS) and an increased state of immunosuppression—such as severe IgG hypogammaglobulinemia and impaired innate immunity—are associated with a dismal outcome in post-transplant IPA.^34^, ^35^, ^36^ In addition, PK factors such as suboptimal oral absorption or poor diffusion into the site of infection (i.e., pulmonary cavitary lesions or through the blood-brain barrier) most likely contribute to the therapeutic failure in LuT recipients treated with ISA.

Our study has some limitations to be acknowledged, including the absence of a control group of LuT recipients receiving VORI, posaconazole or L-AmB. Although the research was originally conceived as prospective, the lower-than-expected recruitment rate forced us to include retrospective cases from one of the centers. Due to the multicenter and ambispective design, some level of underreporting of trAEs must be considered. Nonetheless, it is reasonable to assume that any potentially missed toxicity would have been mild in severity and not led to the premature discontinuation of ISA. A proportion of patients lacked appropriate respiratory tract sampling or thoracic imaging at EoT to evaluate mycological and radiological responses. Moreover, the duration of follow-up (with a median of around 100 days) could have been insufficient to fully capture late relapse of *Aspergillus* infection. Finally, attributable mortality was established according to investigator criteria at each center, introducing potential for misclassification bias. On the other hand, strengths comprise the large number of LuT recipients, the granularity of data collected through the 2 weeks following EoT, and the representativeness and external validity of a multicenter cohort recruited between 2019 and 2024.

In conclusion, ISA therapy was well tolerated in this multicenter study—including the largest number to date of LuT recipients treated with ISA—and was associated with lower rates of trAEs and premature discontinuation than those reported for VORI in previous studies. Our experience suggests that DDIs with tacrolimus are safely manageable in daily clinical practice through adequate TDM. These findings contribute to the still-limited body of evidence on the role of ISA for treating invasive *Aspergillus* syndromes in the LuT population.

## Author contributions

J.T.S., F.L.M., M.F.R., and J.M.A.: conception and design of the study; J.T.S., A.d.P., A.G.V., A.F.C., A.C., E.R.A., J.T.C., A.M., C.B., O.L., D.B.N., D.S.R., and M.A.P.: acquisition of data; J.T.S. and M.F.R.: analysis and interpretation of data; J.T.S.: preparing of the first draft; F.L.M., M.F.R., and J.M.A.: revision of the first draft; all authors: revision of the second draft, commented of the draft, and have read and approved the final submitted version.

## Funding

This research was supported by an unrestricted grant from 10.13039/100004319Pfizer Spain (grant no. WI243034). The funding sources had no role in study design, data collection and analysis, interpretation of data, manuscript preparation, or decision to submit. The corresponding author had full access to all the data in the study and had final responsibility for the decision to submit it for publication.

## Declaration of generative AI and AI-assisted technologies in the writing process

During the preparation of this work, the author used ChatGPT in order to check the spelling and improve the readability of the abstract section. After using this tool/service, the author reviewed and edited the content as needed and takes full responsibility for the content of the published article.

## Declaration of Competing Interest

The authors declare the following financial interests/personal relationships which may be considered as potential competing interests: Jose Maria Aguado reports financial support was provided by Pfizer Spain (grant no. WI243034). Jose Tiago Silva reports a relationship with Pfizer that includes funding grants. The other authors declare that they have no known competing financial interests or personal relationships that could have appeared to influence the work reported in this paper.

## Appendix A Supplementary data

Table S1. Demographics, Clinical Characteristics, Treatment-related Variables and Outcomes According to the Form of Recruitment (Retrospective and Prospective) in the Cohort Study

.

Table S2. Comparison Between the Most Relevant Results From the Present Study and the Two Previous Studies That Have Analyzed the Effectiveness and Safety of ISA in SOT Recipients

## Footnotes

Appendix A Supplementary data associated with this article can be found in the online version at doi:10.1016/j.jhlto.2025.100419.
