# Supplementary material for: Experience with isavuconazole in lung transplant recipients with Aspergillus isolation in respiratory tract specimens: A multicenter, observational study
Source: JHLT Open. 2025 Oct 29;11:100419. doi: 10.1016/j.jhlto.2025.100419 (PMC12671346; doi:10.1016/j.jhlto.2025.100419)
Supplement: Supplementary file 1 — Table S1. Demographics, Clinical Characteristics, Treatment-related Variables and Outcomes According to the Form of Recruitment (Retrospective and Prospective) in the Cohort Study [file mmc1.docx]

**Supplementary Material**

**Table S1.** Demographics, clinical characteristics, treatment-related variables and outcomes according to the form of recruitment (retrospective and prospective) in the cohort study.

| **Variable** | **Retrospective**  (n = 20) | **Prospective**  (n = 30) | ***P-*value** |
| --- | --- | --- | --- |
| Age at transplantation, years [mean ± SD] | 57.0 ± 10.4 | 56.3 ± 13.5 | 0.842 |
| Age at initiation of ISA therapy, years [mean ± SD] | 59.9 ± 8.8 | 58.1 ± 10.9 | 0.531 |
| Male gender [n (%)] | 16 (80) | 23 (76.7) | 1.000 |
| Underlying end-stage lung disease [n (%)] |  |  | 0.260 |
| COPD | 9 (45.0) | 16 (53.3) |  |
| Usual interstitial pneumonia | 7 (35.0) | 4 (13.3) |  |
| Primary pulmonary hypertension | 1 (5.0) | 5 (16.7) |  |
| Interstitial lung disease | 1 (5.0) | 4 (13.3) |  |
| Cystic fibrosis | 1 (5.0) | 1 (3.3) |  |
| Bilateral bronchiectasis | 1 (5.0) | 0 (0.0) |  |
| Pre-transplant *Aspergillus* colonization [n (%)] | 2 (10.0) | 2 (6.7) | 1.000 |
| Type of LuT [n (%)] |  |  | 0.236 |
| Single lung | 7 (35.0) | 6 (20.0) |  |
| Double lung | 13 (65) | 24 (80.0) |  |
| Previous LuT [n (%)] | 0 (0.0) | 1 (3.3) | 1.000 |
| Pre-transplant comorbidities [n (%)] |  |  |  |
| Diabetes mellitus | 6 (30.0) | 5 (16.7) | 0.488 |
| Chronic heart disease | 4 (20.0) | 3 (10.0) | 0.416 |
| Post-transplant complications [n (%)] |  |  |  |
| Reintervention within the first month | 5 (25.0) | 4 (13.3) | 0.460 |
| Acute graft rejection | 9 (45.0) | 5 (16.7) | 0.029 |
| CLAD | 3 (15.0) | 1 (3.3) | 0.289 |
| Post-transplant antifungal prophylaxis [n (%)] | 18 (90.0) | 29 (96.7) | 0.556 |
| Induction therapy with basiliximab [n (%)] | 17 (85.0) | 18 (60.0) | 0.059 |
| Immunosuppressive regimen at initiation of ISA [n (%)] |  |  |  |
| Corticosteroids | 19 (95.0) | 30 (100.0) | 0.400 |
| Daily prednisone dose or equivalent, mg [median (IQR)] | 30.0 (15.0 – 60.0) | 30.0 (10.0 – 40.0) | 0.286 |
| Tacrolimus | 19 (95.0) | 29 (96.7) | 1.000 |
| Trough level, mg/mL [median (IQR)] | 9.5 (6.1 – 11.3) | 11.2 (8.6 – 13.7) | 0.062 |
| Mycophenolate mofetil or mycophenolate sodium | 10 (50.0) | 20 (66.7) | 0.239 |
| Daily dose, g [median (IQR)] | 2.0 (1.4 – 2.0) | 2.0 (1.5 – 2.0) | 0.585 |
| Azathioprine | 0 (0.0) | 1 (3.3) | 1.000 |
| Indication for antifungal therapy [n (%)] |  |  | 0.087 |
| IPA | 10 (50.0) | 8 (26.7) |  |
| TBA | 7 (35.0) | 20 (66.7) |  |
| Preemptive therapy | 3 (15.0) | 2 (6.7) |  |
| Time interval from LuT to initiation of ISA therapy, days [median (IQR)] | 356.5 (37.8 – 1093.3) | 184.0 (20.5 – 352.5) | 0.132 |
| Completion rate of the planned course of ISA therapy [n (%)] | 12 (60.0) | 23 (76.7) | 0.228 |
| trAE requiring premature discontinuation [n (%)] | 2 (10.0) | 3 (10.0) | 1.000 |
| Outcomes [n (%)] |  |  |  |
| Clinical response at EoT | 9 / 17 (52.9) | 21 / 28 (75.0) | 0.193 |
| Mycological response at EoT | 10 / 14 (71.4) | 22 / 24 (91.7) | 0.167 |
| Radiological response at EoT | 4 / 6 (66.7) | 5 / 8 (62.5) | 1.000 |
| CLAD: chronic lung allograft dysfunction; COPD: chronic obstructive pulmonary disease; EoT: end of treatment; IPA: invasive pulmonary aspergillosis; IQR: interquartile range; ISA: isavuconazole; LuT: lung transplantation; SD: standard deviation; TBA: tracheobronchial aspergillosis; trAE: treatment-emergent adverse event. | | | |
