# Supplementary material for: Experience with isavuconazole in lung transplant recipients with Aspergillus isolation in respiratory tract specimens: A multicenter, observational study
Source: JHLT Open. 2025 Oct 29;11:100419. doi: 10.1016/j.jhlto.2025.100419 (PMC12671346; doi:10.1016/j.jhlto.2025.100419)
Supplement: Supplementary file 2 — Table S2. Comparison Between the Most Relevant Results From the Present Study and the Two Previous Studies That Have Analyzed the Effectiveness and Safety of ISA in SOT Recipients [file mmc2.docx]

**Supplementary Material**

**Table S2.** Comparison between the most relevant results from the present study and the two previous studies that have analyzed the effectiveness and safety of isavuconazole in solid organ transplant recipients.

|  | **Present study** | **SOTIS Study** [1] | **ISASOT Study** [2] |
| --- | --- | --- | --- |
| Clinical response | 66.7% at EoT | 56.3% at week 12 | 50.9% at EoT |
| Culture conversion | 84.2% | NR | 38.6%^a^ |
| IFI-attributable mortality | 12.0% | 22.2%^b^ | 11.3% |
| trAE | 24.0% | 17.3% | 49.1% |
| trAE requiring discontinuation of therapy | 10.0% | 6.2% | 11.3% |
| EoT: end of treatment; IFI: invasive fungal infection; NR: not reported; trAE: treatment-emergent adverse events.  ^a^ Overall, 20.0% of patients received isavuconazole for an infection not caused by *Aspergillus* spp, and 27.3% had no follow-up cultures performed at EoT  ^b^ The authors did not report separate mortality rates for invasive aspergillosis and mucormycosis (which accounted for 14.8% of patients). | | | |

**References**

1. Fernandez-Ruiz M, Bodro M, Gutierrez Martin I, Rodriguez-Alvarez R, Ruiz-Ruigomez M, Sabe N, et al. Isavuconazole for the Treatment of Invasive Mold Disease in Solid Organ Transplant Recipients: A Multicenter Study on Efficacy and Safety in Real-life Clinical Practice. Transplantation. 2023;107(3):762-773.
2. Monforte A, Los-Arcos I, Martin-Gomez MT, Campany-Herrero D, Sacanell J, Berastegui C, et al. Safety and Effectiveness of Isavuconazole Treatment for Fungal Infections in Solid Organ Transplant Recipients (ISASOT Study). Microbiol Spectr. 2022;10(1):e0178421.
